# Identification of a Sacral, Visceral Sensory Transcriptome in Embryonic and Adult Mice

**DOI:** 10.1523/ENEURO.0397-19.2019

**Published:** 2020-02-19

**Authors:** C. J. A. Smith-Anttila, E. A. Mason, C. A. Wells, B. J. Aronow, P. B. Osborne, J. R. Keast

**Affiliations:** 1 Department of Anatomy and Neuroscience, University of Melbourne, Parkville, 3010 Victoria, Australia; 2 Department of Pediatrics, College of Medicine, University of Cincinnati, Department of Biomedical Informatics, Cincinnati Children’s Hospital Medical Center, Cincinnati, OH 45229

**Keywords:** autonomic regulation, nociceptor, pelvic pain, sexual dimorphism, urogenital, visceral pain

## Abstract

Visceral sensory neurons encode distinct sensations from healthy organs and initiate pain states that are resistant to common analgesics. Transcriptome analysis is transforming our understanding of sensory neuron subtypes but has generally focused on somatic sensory neurons or the total population of neurons in which visceral neurons form the minority. Our aim was to define transcripts specifically expressed by sacral visceral sensory neurons, as a step towards understanding the unique biology of these neurons and potentially leading to identification of new analgesic targets for pelvic visceral pain. Our strategy was to identify genes differentially expressed between sacral dorsal root ganglia (DRG) that include somatic neurons and sacral visceral neurons, and adjacent lumbar DRG that comprise exclusively of somatic sensory neurons. This was performed in adult and E18.5 male and female mice. By developing a method to restrict analyses to nociceptive Trpv1 neurons, a larger group of genes were detected as differentially expressed between spinal levels. We identified many novel genes that had not previously been associated with pelvic visceral sensation or nociception. Limited sex differences were detected across the transcriptome of sensory ganglia, but more were revealed in sacral levels and especially in Trpv1 nociceptive neurons. These data will facilitate development of new tools to modify mature and developing sensory neurons and nociceptive pathways.

## Significance Statement

In this study of mouse dorsal root ganglia (DRG), we have identified numerous features of sensory neurons that vary between lumbar and sacral spinal levels and that are potentially involved in unique physiology and pathophysiology of visceral sensation and pain. We further identify maturational components of this sacral visceral transcriptome by comparing data from embryonic and adult mice. There are limited sex differences across the transcriptome of embryonic or adult sensory ganglia, but in adults these can be revealed in sacral levels and especially in Trpv1 nociceptive neurons. These datasets will encourage identification of new tools to modify mature or developing sensory neurons and adult nociceptive pathways.

## Introduction

Persistent pain associated with the pelvic organs, such as bladder pain syndrome/interstitial cystitis, irritable bowel syndrome and endometriosis, is difficult to treat (Udoji and Ness, 2013; Origoni et al., 2014; Kim and Han, 2018; Grundy et al., 2019; Zheng et al., 2019). These conditions do not respond reliably to analgesics that are commonly administered for somatic pain, indicating that distinct mechanisms underlie their pathobiology. Healthy visceral sensory neurons also have distinct functional properties that underlie encoding of a wide range of changes within organs (Robinson and Gebhart, 2008; Sikandar and Dickenson, 2012; Schwartz and Gebhart, 2014; Gebhart and Bielefeldt, 2016). We are unaware of many of these internal changes, contrasting with our conscious awareness of somatic sensations such as heat, cold, touch, and vibration. Understanding the molecular signature underlying the specific functional features of healthy visceral sensory neurons may reveal sites of vulnerability during disease and targets for therapeutic development.

We set out to define the unique biology of visceral sensory neurons innervating pelvic organs in adult mice. In rodents, the majority are located in dorsal root ganglia (DRG) in spinal levels lumbar 6 and sacral 1 (L6-S1; Dang et al., 2005; Xu and Gebhart, 2008; Forrest et al., 2013); we refer to these as “sacral” DRG as they are functionally equivalent to neurons found exclusively in sacral levels in humans. Pelvic visceral sensory neurons comprise two major classes, those with lightly myelinated (A-δ) or unmyelinated (C) axons (Dang et al., 2005; Franken et al., 2014; de Groat et al., 2015; Gebhart and Bielefeldt, 2016; Brierley et al., 2018). Many express transient receptor potential (TRP) channels that transduce noxious stimuli, including Trpv1, Trpa1, and Trpm8 (Skryma et al., 2011; Franken et al., 2014; Merrill et al., 2016), which are also expressed in subpopulations of somatic nociceptors (Patapoutian et al., 2009; Julius, 2013). In the L6-S1 DRG, the visceral sensory neurons are greatly outnumbered by sensory neurons innervating somatic targets in the lower body (Robinson and Gebhart, 2008). Therefore, to reveal the molecular profile of the visceral sensory neuron population innervating the sacral region we compared the L6-S1 DRGs with the adjacent ganglia located at lumbar 4 and 5 (L4-5; “lumbar”) housing exclusively somatic sensory neurons. Genes differentially expressed between the lumbar and sacral groups were considered to be largely driven by the population of visceral neurons present only in the sacral region. A similar analysis was performed in DRG dissected from E18.5 mouse embryos, aiming to reveal transient pelvic visceral features and those emerging prior to organ maturation. To define the molecular profile of pelvic visceral nociceptors we developed a novel method for aggregating DRG neurons expressing transcripts for Trpv1 that has a prominent role in the detection and transduction of nociceptive stimuli (Patapoutian et al., 2009; Julius, 2013). To our knowledge, only one prior transcriptome study has specifically investigated pelvic visceral sensory neurons (Hockley et al., 2019). This study focused on intestinal innervation and was performed using a single cell approach to characterize neurons in thoraco-lumbar or lumbosacral DRGs retrogradely labeled from the distal colon in adult male mice.

All studies were performed in both sexes to identify genes that may underpin aspects of commonly reported sex differences in the propensity to develop pelvic pain states (Mogil, 2012; Bartley and Fillingim, 2013). We predicted that our outcomes may differ from a recent study (Lopes et al., 2017b) that identified very few differences between the male and female DRG transcriptome but which studied lumbar DRG that do not have as pronounced innervation of sexually dimorphic tissues.

Several studies have characterized the transcriptome of different sensory neuron populations in rodent DRG (Lerch et al., 2012; Manteniotis et al., 2013; Chiu et al., 2014; Goswami et al., 2014; Thakur et al., 2014; Reynders et al., 2015; Usoskin et al., 2015; Lopes et al., 2017a; Ray et al., 2018), however to our knowledge no studies have adopted our comparative approach to define unique properties of the total population of sacral visceral sensory neurons, in both adult and embryonic DRG and in both sexes. In this study, we identify many novel genes not previously associated with pelvic visceral sensation or nociception and provide the scientific community with a rich data resource to identify many more relevant patterns of gene expression.

## Materials and Methods

### Animals

All procedures complied with the Australian Code for the Care and Use of Animals for Scientific Purposes (National Health and Medical Research Council of Australia) and were approved by the Animal Ethics Committee at the University of Melbourne.

Adult male and female C57Bl/6 mice (six to eight weeks) were purchased from the Animal Resources Centre (ARC). Embryos (E18.5) were obtained from pregnant female C57Bl/6 mice purchased from ARC and housed until the appropriate stage. Heterozygous TrpV1^PLAP-nLacZ^ mice originally generated and validated by Cavanaugh et al. (2011b) were purchased from JAX (B6.129-*Trpv1^tm2Bbm^*/J, stock #017623) and maintained as a homozygous line.

Mice were housed in Sealsafe Plus individually ventilated cages (Tecniplast Australia Pty Ltd) with autoclaved recycled paper bedding (FibreCycle Pty Ltd). The temperature was maintained at 21°C (range 19–24°C) and the humidity at 40%. A 12/12 h light/dark cycle was used, with lights on at 7 A.M. The animals had *ad libitum* access to food and filtered water.

### Tissue removal and RNA purification

DRG from spinal levels L4-5 and L6-S1 were dissected from five male and five female E18.5 embryos and placed immediately in Hibernate E medium (Thermo Fisher Scientific, Life Technologies) on ice until all dissections were complete. Ganglia were then transferred to 350-μl RLT Plus buffer (QIAGEN) with 20-μl 2 M DTT and homogenized by passing through a 25-gauge needle seven to eight times on ice.

For adult studies, L4-5 and L6-S1 DRG were dissected from five male and seven female C57Bl/6 mice (six to eight weeks) and immediately placed into 1× Tyrode’s solution (130 mM NaCl, 20 mM NaHCO_3_, 10 mM HEPES, 3 mM KCl, 4 mM CaCl_2_, 1 mM MgCl_2_, 12 mM glucose, and 0.5× antibiotic/antimycotic solution; Thermo Fisher Scientific, Life Technologies) on ice. The nerve roots were then removed and the DRG transferred to 350-μl RLT Plus buffer (QIAGEN) with 20-μl 2 M DTT and homogenized by passing through a 21-gauge needle three to four times, followed by a 25-gauge needle seven to eight times on ice.

Following homogenization, total RNA for E18.5 embryos and adult mice was extracted using the QIAGEN RNeasy Plus Micro kit (QIAGEN) according to the manufacturer’s instructions. RNA quality was analyzed using the Agilent 2200 TapeStation (Agilent Technologies) for adult DRG and the Agilent 2100 Bioanalyzer (Agilent Technologies) for E18.5 samples. RIN numbers were routinely more than eight.

### Validation of methodology to visualize Trpv1 expression in living neurons

We developed a new approach for aggregating neurons expressing Trpv1 rather than isolating neurons from a Trpv1-Cre mouse. This was done because Trpv1 is downregulated in many sensory neurons during development, therefore DRG isolation from the Trpv1-Cre mouse would identify the full Trpv1 lineage rather than exclusively adult expression (Cavanaugh et al., 2011a; Goswami et al., 2014).

Lumbosacral DRG were dissected from adult (six to eight week) male and female Trpv1^PLAP-nLacZ^ homozygous mice, and the ganglia placed into 1× Tyrode’s solution immediately after dissection, as described above. DRG were digested in 0.93 mg/ml collagenase type 1 (Worthington Biochemical Corporation) in 1× Tyrode’s solution at 37°C for 1 h followed by 0.93 mg/ml collagenase with 0.03 mm trypsin-EDTA (Thermo Fisher Scientific, Invitrogen) in 1× Tyrode’s solution for 1 h at 37°C. DRG were washed twice in Leibovitz’s L-15 medium (L-15 medium; Life Technologies) then triturated with a P-1000 pipette in L-15 medium. Cells were centrifuged at 300 × *g* at 4°C and resuspended in L-15 medium; 1 mM DDAO galactoside [9*H*-(1,3-dichloro-9,9-dimethylacridin-2-one-7-yl) β-D-galactopyranoside; DDAOG; Thermo Fisher Scientific, Molecular Probes, catalog #D6488] in DMSO was added to the cell suspension at a final concentration of 0.01 mm DDAOG and incubated on ice for 30 min. DDAOG is cleaved by β-galactosidase (Lindvall et al., 2009) in the Trpv1 cells to produce 7-hydroxy-9H(I,3-dichloro-9,9-dimethylacridin-2-one (DDAO). The reaction was stopped by the addition of 1.0 mm EDTA in L-15, followed by centrifuging at 300 × *g* at 4°C then cells resuspended in L-15. The dissociated cells were plated onto poly-ornithine-coated coverslips and incubated for 1 h at 37°C in 5% CO_2_ to allow the cells to adhere. The cells were then fixed for 30 min in 4% PFA and processed for immunohistochemical localization of β-galactosidase. This comprised: washing in 0.1 M PBS (pH 7.2), blocking for 1 h in 10% non-immune horse serum with 0.1% Triton X-100 in PBS, washing in PBS then incubating with anti-β-galactosidase (1:10,000; MP-Biomedicals, Thermo Fisher Scientific; catalog #559761, lot #7059; RRID: AB_2687418) for 2 h at room temperature. Cells were then washed in PBS, incubated with donkey anti-rabbit AF594 (1:1000; Jackson ImmunoResearch; catalog #711–585-152) for 1 h at room temperature, washed and then mounted on slides with buffered glycerol. Images of DRG neurons were captured with a Zeiss AxioImager M2 microscope and AxioCam MRm camera using Zen 2 blue edition software (Carl Zeiss). Minimal linear adjustment of contrast and brightness (Adobe Creative Suite) was performed uniformly across images to produce figures that most closely resembled the labeling as viewed down the microscope.

### DRG dissociation and flow cytometry to isolate Trpv1 neurons

DRG (L4-5 and L6-S1) were dissected from five male and five female adult (six to eight week) Trpv1^PLAP-nLacZ^ homozygous mice and placed into 1× Tyrode’s solution on ice, dissociated and treated with DDAOG, as described above. Following incubation with DDAOG, cells were centrifuged at 200 × *g* at 4°C for 5 min and resuspended in 500-μl L-15 medium. Cells were filtered through a 40-μm cell strainer, 200 ng/ml DAPI added as dead cell stain and the cell suspension immediately evaluated by flow cytometry.

Cells were sorted on a Becton Dickinson FACSAria III (100 μm at 20 psi with a 1.5 ND filter). The brightest 15% of DDAO-positive cells were sorted into 500-μl RLT Plus buffer (QIAGEN) with 120 mm DTT. This cell fraction was then homogenized by passing it through a 25-gauge needle three to four times on ice and the RNA extracted immediately using the Qiagen RNeasy Plus Micro kit (QIAGEN) according to the manufacturer’s instructions. RNA quality was analyzed using the Agilent 2100 Bioanalyzer (Agilent Technologies).

Age matched C57Bl/6 mice were used as negative and DDAOG positive controls for all flow cytometry experiments. DRG from all relevant spinal levels were collected and processed in the same way as the cells from the Trpv1^PLAP-nLacZ^ homozygous mice.

### RNA sequencing (RNA-seq)

Total RNA was amplified to create double stranded cDNA using the Ovation RNA-Seq System v2 (NuGEN) according to the manufacturer’s protocol. cDNA concentrations were measured using the Qubit dsDNA BR assay, with cDNA size determined by using a DNA 1000 Chip. Libraries were then prepared with the Nextera XT DNA Sample Preparation kit (Illumina Technologies). The purified cDNA was captured on an Illumina flow cell for cluster generation. Libraries were sequenced on the Illumina HiSeq2500 following the manufacturer’s protocol. The concentration of the pool is optimized to acquire at least 15–20 million reads per sample.

### Public hosting of data

Data are hosted as three separate experiments: Adult, Trpv1, E18.5. All raw and normalized RNA-seq data are available at GEO under accession number GSE131623. To increase rigor, reproducibility and transparency, raw data generated as part of this study were deposited into the GUDMAP (the NIH-sponsored Genitourinary Development Molecular Anatomy Project) consortium database (www.gudmap.org) and are fully accessible at https://doi.org/10.25548/16-WK64. Stemformatics (www.stemformatics.org) provides an additional interface to interact with the data directly and a data portal to download either normalized or raw data associated with each experiment.

### Data processing

For all experiments, FASTQ libraries were quality checked using FASTX toolkit version 0.0.14 and were aligned to the mm9 Ensembl 67 genome retaining uniquely mapping reads only. Samples were aligned using Subread (version 1.18 or higher) Bioconductor package for R (version 3.2.1 or higher) with max indels = 5, max mismatches = 5. Raw reads were counted and annotated against Ensembl v67 genes using *featureCounts* function of package Subread. Gene counts were normalized using the Voom method of limma package v3.26.9 under R v3.2.2 with additional TMM library size adjustment using edgeR package v3.12.1 and further transformation to produce RPKM log2 abundance measures. We observed a technical batch effect between the two experimental batches in the adult dataset: five samples from batch 1 and 19 samples from batch 2. This was corrected on the RPKM log2 expression data using *removeBatchEffect* function of limma package v3.26.9.

### Experimental design and statistical analysis

This study comprised three separate experimental datasets, analyzing the transcriptome from (1) the entire population of cells in adult DRG from selected spinal levels; (2) the subpopulation of cells isolated on the basis of Trpv1 expression in these selected spinal levels of adult DRG; and (3) the entire population of neurons in these spinal levels of DRG in the late embryo (E18.5). In each dataset, we sought to detect effects of spinal level or sex, or an interaction between the two factors.

For Embryo and Trpv1 datasets, this differential expression analysis was performed using the Voom method of limma v3.30.13 under R v3.3.2. We supplied raw counts to Voom as required by the method (Voom performs TMM library-size normalization and log2 transformation by default, in addition to its moderation of linear modelling parameters). Donor blocking was included in the DE model. Genes not expressing at least 1 count per million in a sample group were removed prior to DE analysis. Genes were defined as differentially expressed when the Benjamini–Hochberg adjusted *p* value was less than or equal to 0.05, reflecting a false discovery rate of 5%. An additional step was performed for the Adult dataset, where it was necessary to remove an experimental batch effect (see below) prior to analysis of differential gene expression. PCA was generated using *prcomp* function from stats package for R 3.5.1. Gene set enrichment analysis was performed using the EGSEA package (Alhamdoosh et al., 2017) under Bioconductor 3.9 for R 3.5.1.

All of the Embryo and Trpv1 RNAseq libraries were prepared and run in the same facility; however, a subset of the adult series was conducted in a second research facility. This difference in site caused a technical or “batch” effect on the data, therefore library source was identified and corrected with the *removeBatchEffect* function from R limma package (Ritchie et al., 2015). In this method, we fitted a linear model (capturing both phenotype and batch dimensions) to the TMM normalized RPKM log2 data and the batch component was subsequently subtracted.

## Results

### Adult DRG containing pelvic viscera-projecting neurons have a distinct transcriptional profile

**Figure 1.**
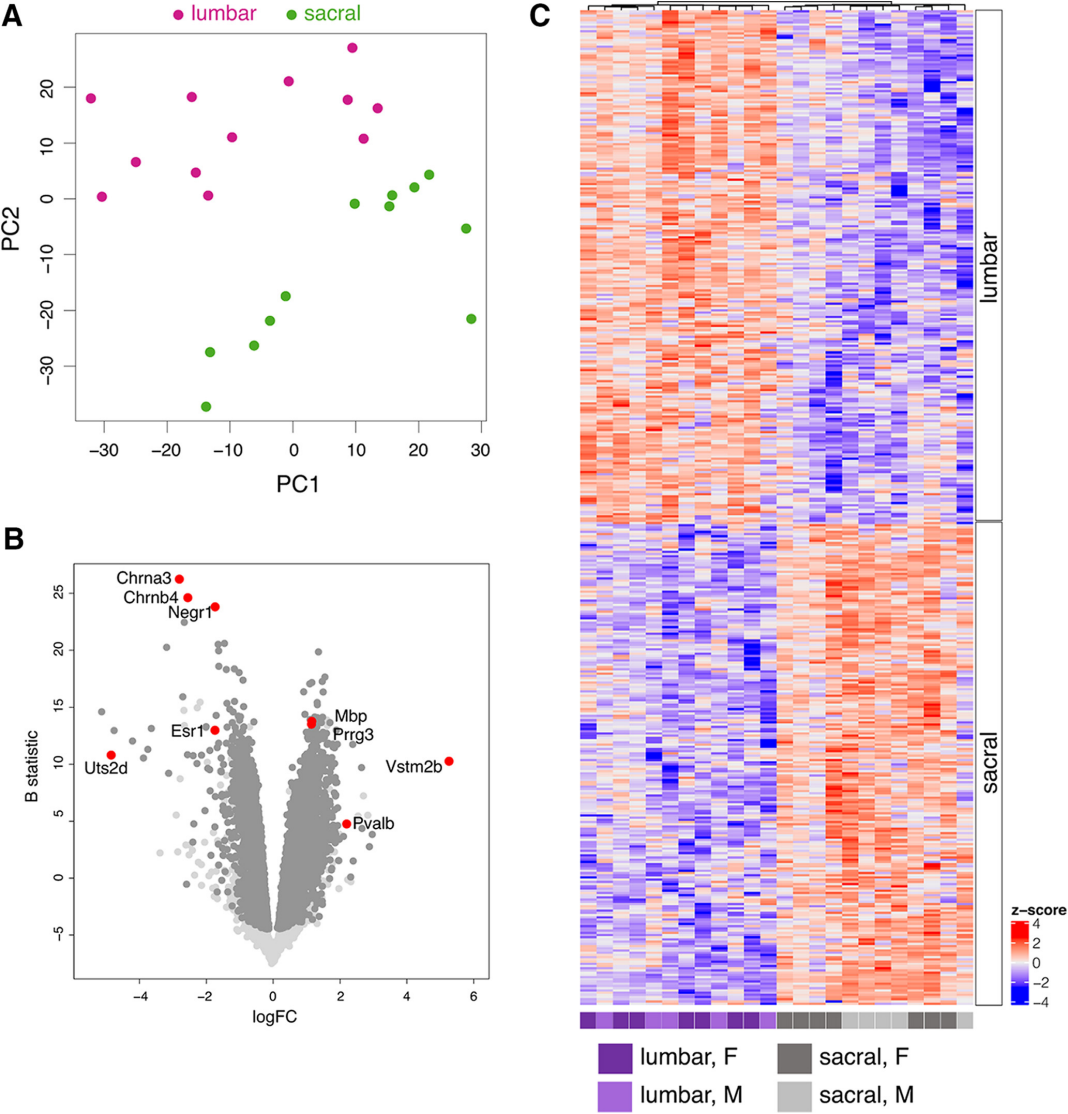
Analysis of DRG from two different spinal levels (lumbar: L4-5; sacral: L6-S1) taken from five male and seven female adult C57Bl/6 mice. ***A***, Principal component analysis (*N* = 23 168; all detected genes) indicates spinal region as the major source of variance in the data. ***B***, Volcano plot illustrates differential expression between lumbar and sacral DRG. Light gray points are genes not differentially expressed, dark gray points are differentially expressed at adjusted *p* <* *0.05; examples of specific differentially expressed genes are highlighted in red. A negative value for the FC indicates an upregulation in sacral DRG, whereas a positive FC indicates upregulation in lumbar DRG. Full dataset are provided in Extended Data Figure 1-1. ***C***, Heatmap with hierarchical cluster for all differentially expressed genes (*N* = 466; adjusted *p *<* *0.05) for adult lumbar and sacral samples (*N* = 12). Both samples and rows are clustered using Pearson correlation. Heat color reflects row-wise *z* score, and samples are colored according to spinal level and sex. Ranked gene lists for heat map are provided in Extended Data Figure 1-2.

The major goal of our study was to identify a distinct transcriptional profile of pelvic visceral sensory neurons that are aggregated in sacral DRG. To evaluate these differences, we pooled RNA-seq data collected from the lumbar and sacral regions of five male and seven female C57Bl/6 adult mice. Principal component analysis (*N* = 23 168; all detected genes) revealed that spinal region was the major source of variance in the data (Fig. 1*A*). Differential expression analysis identified 466 genes significantly different between lumbar and sacral DRG (Fig. 1*B*,*C*; Extended Data Figs. 1-1, 1-2; adjusted *p *<* *0.05). Many of the genes upregulated in lumbar DRG (e.g., Nefh, Pvalb, Ntrk3, Runx3) are characteristic of large, myelinated sensory neurons (including large proprioceptive neurons; Usoskin et al., 2015). We expected these to comprise a higher proportion of neurons in lumbar than in the sacral DRG, as the latter include viscera-projecting neurons. Conversely, many of the genes upregulated in our sacral DRG dataset are characteristic of small, unmyelinated sensory neurons (Usoskin et al., 2015) expected to comprise a higher proportion of neurons here than in the purely somatic lumbar DRG. These genes upregulated in sacral DRG include: the nociceptor transducer, Trpv1; the neuropeptides and precursor peptides, Tac1 (preprotachykinin), Adcyap1 (PACAP), and Cartpt; and Esr1 (estrogen receptor α). Homeobox genes Hoxc8 and Hoxb9 showed upregulated expression in lumbar DRG, whereas Hoxc10 and Hoxd11 were upregulated in sacral DRG; this further validated our identification of distinct DEGs at different spinal levels.

10.1523/ENEURO.0397-19.2019.f1-1 Extended Data Figure 1-1 Differential expression analysis for all genes detected in adult lumbar versus sacral DRG samples. A negative value for the FC indicates an upregulation in sacral DRG, whereas a positive FC indicates upregulation in lumbar DRG. Download Figure 1-1, XLSX file.

**Figure 2.**
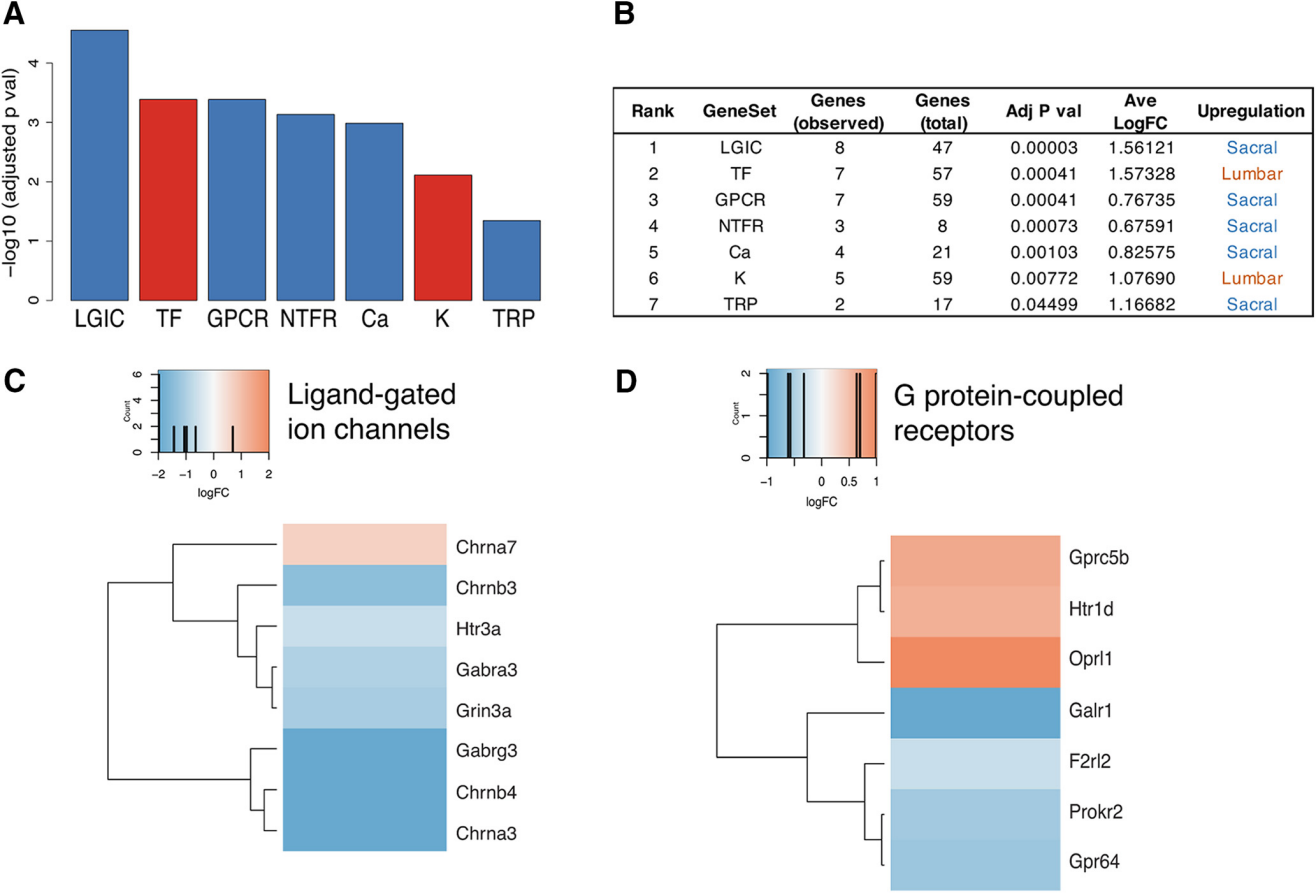
Gene set enrichment analysis of DRG neurons taken from five male and seven female adult C57Bl/6 mice, comparing expression at lumbar (L4-5) and sacral (L6-S1) levels. Seven of 10 gene sets (grouped according to shared function; see Extended Data Fig. 2-1) were enriched (adjusted *p *<* *0.05): LGICs, TFs, GPCRs, NTFRs, Ca, and K channels, and TRP channels. ***A***, ***B***, Five gene classes showed upregulation in sacral DRG (blue) and two showed upregulation in lumbar DRG (red). ***C***, ***D***, Genes differentially expressed between lumbar and sacral spinal levels from the LGIC and GPCR classes. Blue indicates upregulation in sacral DRG and red upregulation in lumbar DRG. Full list of differentially genes identified by this analysis is provided in Extended Data Figure 2-2, and complete set of heatmaps is provided in Extended Data Figure 2-3.

10.1523/ENEURO.0397-19.2019.f1-2 Extended Data Figure 1-2 Ranked gene lists corresponding to heat maps (adult, Trpv1, embryo). Each list comprises the differentially expressed genes plotted in hierarchically clustered heatmaps for adult (Fig. 1*C*), Trpv1 (Fig. 4*D*), and embryo (Fig. 6*D*) datasets. Download Figure 1-2, XLSX file.

Given that evaluating molecular profiles associated with pelvic visceral pain lies at the heart of our investigation, we curated a set of 298 genes strongly relevant to neural signaling, nociceptor sensitization or specification. Genes were arranged into 10 individual sets according to function: calcium (Ca) channels, chloride (Cl) channels, G-protein-coupled receptors (GPCRs), ligand-gated ion channels (LGICs), neurotrophic factor receptors (NTFRs), neurotrophic factors (NTFs), potassium (K) channels, sodium (Na) channels, transcription factors (TFs), and TRP channels. Gene set enrichment analysis revealed that seven of 10 gene sets were enriched overall (Fig. 2; Extended Data Figs. 2-1, 2-2, 2-3; adjusted *p *<* *0.05). Specifically, TF and K channels were upregulated in lumbar DRG, while LGIC, GPCR, NTFR, Ca, and TRP channels were upregulated in sacral DRG. One of the genes in the LGIC group (Htr3a) has been previously implicated in bladder sensory dysfunction (Ritter et al., 2017), but to our knowledge the various glutamate and GABA receptor transcripts upregulated in sacral DRG (Grin3a, Gabra3, Gabrg3) have not yet been examined in this context. LGIC receptors for the neurotransmitter acetylcholine showed a diverse expression pattern, with Chrna3 and Chrnb4 upregulated in sacral DRG, Chrna7 upregulated in lumbar DRG and other subunits not differentially expressed between spinal levels. This indicates potentially complex and tissue-specific mechanisms by which acetylcholine can modulate sensation. Several genes upregulated in sacral DRG, including Galr1, Gap43, Mmp8, and Negr1, are implicated in axonal growth and remodeling of sensory neurons, a property also relevant to inflammatory-induced or injury-induced pain. The potent vasoconstrictor urotensin 2d (Uts2d; Chatenet et al., 2013) displayed one of the highest fold-change (FC = 4.85) of genes upregulated in sacral ganglia.

10.1523/ENEURO.0397-19.2019.f2-1 Extended Data Figure 2-1 Curated gene sets associated with neuronal signaling, nociceptor sensitization or specification. This list of genes is the focus of gene set enrichment analyses illustrated in Figures 2, 5. Download Figure 2-1, XLSX file.

10.1523/ENEURO.0397-19.2019.f2-2 Extended Data Figure 2-2 Gene set enrichment analyses for Adult and Trpv1 DRG. From the full curated gene set (Extended Data Fig. 2-1), these genes were identified as differentially expressed between lumbar and sacral DRG for the total adult or the Trpv1 adult dataset. These genes are illustrated in Figures 2, 5 and Extended Data Figures 2-3, 5-1. Download Figure 2-2, XLSX file.

**Figure 3.**
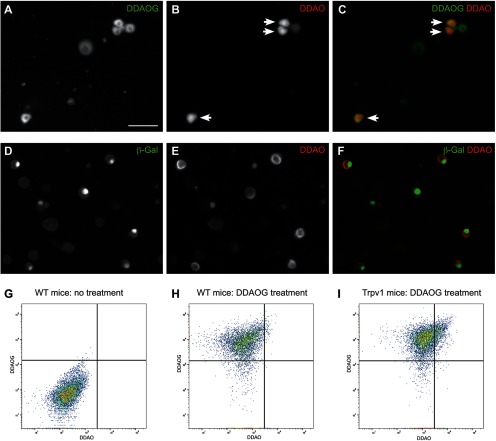
Strategy for isolation of Trpv1-expressing neurons in mouse DRG. ***A*–*F***, Neurons isolated from lumbosacral adult DRG of TrpV1^PLAP-nLacZ^ mice were incubated with the fluorescent galactosidase substrate, DDAOG (excitation/emission maxima ∼460/610 nm). ***A***, Five neurons that have taken up DDAOG. ***B***, Three neurons (arrows) are visible by their fluorescent hydrolysis product (excitation/emission maxima ∼645/660), also evident in the nucleus, so deduced to express galactosidase, i.e., Trpv1 neurons. ***C***, Merge of panels ***A***, ***B***. ***D***, Neurons expressing β-galactosidase visualized using immunohistochemistry; expression is restricted to the nucleus. ***E***, Each neuron in the field has converted DDAOG to DDAO. ***F***, Merge of panels ***D***, ***E***. Bar in ***A*** applies to all micrographs and represents 50 μm. ***G*–*I***, Representative outputs from flow cytometry of mouse lumbosacral DRG. ***G***, Neurons isolated from wild-type mice were not treated with DDAOG. ***H***, Neurons isolated from wild-type mice were treated with DDAOG, but without expressing β-galactosidase cannot hydrolyze this to form DDAO. ***I***, Neurons isolated from of TrpV1^PLAP-nLacZ^ mice were treated with DDAOG; many cells have hydrolyzed DDAOG to DDAO (i.e., Trpv1 neurons) and others have taken up DDAOG but not hydrolyzed this to DDAO (i.e., Trpv1-negative neurons).

10.1523/ENEURO.0397-19.2019.f2-3 Extended Data Figure 2-3 Gene set enrichment analysis of DRG neurons taken from five male and seven female adult C57Bl/6 mice, comparing expression at lumbar (L4-5) and sacral (L6-S1) levels. Seven of 10 gene sets (grouped according to shared function; see Extended Data Fig. 2-1) were enriched (adjusted *p *<* *0.05): LGICs, TFs, GPCRs, NTFRs, Ca and K channels, and TRP channels. Heatmaps show genes differentially expressed between lumbar and sacral spinal levels from each class. Blue indicates upregulation in sacral DRG and red upregulation in lumbar DRG. A list of genes identified by this analysis is provided in Extended Data Figure 2-2. Download Figure 2-3, PDF file.

### Isolation of Trpv1 neurons provides a higher resolution identification of genes differentially expressed between lumbar and sacral DRG

To isolate sensory neurons expressing Trpv1, we dissected DRG from relevant spinal levels of previously characterized adult Trpv1^PLAP-nLacZ^ mice. The neurons were dispersed into a single cell suspension, then treated with DDAOG that is cleaved to DDAO exclusively in the β-galactosidase expressing Trpv1 neurons; these neurons can then be detected and isolated by flow cytometry.

We first confirmed that in DRG suspensions derived from these mice, DDAO was produced exclusively in a subset of neurons (Fig. 3*A–C*) immunoreactive for β-galactosidase, i.e., Trpv1-expressing neurons (Fig. 3*D–F*). We then used flow cytometry to isolate the brightest 15% of DDAO-positive neurons (Fig. 3*G–I*), to obtain a Trpv1-enriched population.

**Figure 4.**
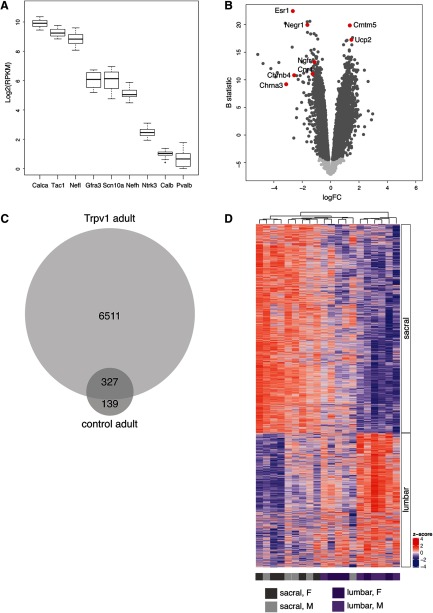
Analysis of neurons isolated by flow cytometry to enrich the Trpv1 population from adult mice (five male, five female). ***A***, Box plot of key markers associated with unmyelinated or myelinated DRG neurons; *x*-axis reflects gene of interest, *y*-axis reflects Log2(RPKM) gene expression. ***B***, Volcano plot illustrating genes differentially expressed between Trpv1 lumbar and sacral DRG neurons. Light gray points are genes not differentially expressed, dark gray points are differentially expressed at adjusted *p *<* *0.05; examples of specific differentially expressed genes are highlighted in red. A negative value for the FC indicates an upregulation in sacral DRG, whereas a positive FC indicates upregulation in lumbar DRG. Full dataset provided in Extended Data Figure 4-1. ***C***, Proportional Venn diagram showing the number of genes differentially expressed between spinal levels, in the total adult DRG population and adult Trpv1 neurons. A total of 466 genes were differentially expressed (adjusted *p *<* *0.05) between lumbar and sacral levels in the total population of adult DRG neurons, 327 of which were also detected as differentially expressed between lumbar and sacral levels in adult Trpv1 neurons. An additional 6511 genes were detected as differentially expressed (adjusted *p *<* *0.05) between lumbar and sacral levels when only the Trpv1 neurons were included. Gene lists summarized in Venn diagram are provided in Extended Data Figure 4-2. ***D***, Heatmap with hierarchical cluster for all differentially expressed genes (*N* = 6838; adjusted *p *<* *0.05) for TRPV1 lumbar and sacral samples (*N* = 10). Both samples and rows are clustered using Pearson correlation. Heat color reflects row-wise *z* score, and samples are colored according to spinal level and sex. Ranked gene lists for heat map are provided in Extended Data Figure 1-2.

The molecular profile of the isolated population was consistent with that of Trpv1 expressing neurons. Genes expressed by small, unmyelinated neurons (e.g., Calca, Tac1, Nefl, Gfra3, Scn10a) were enriched and those expressed by large neurons with myelinated axons (e.g., Ntrk3, Calb, Pvalb) were reduced (Fig. 4*A*). Nefh is expressed by both large-diameter heavily myelinated neurons and medium-diameter lightly myelinated neurons (Lawson et al., 1993; Forrest et al., 2013) and showed intermediate expression in the Trpv1 dataset.

10.1523/ENEURO.0397-19.2019.f4-1 Extended Data Figure 4-1 Differential expression analysis for all genes detected in TRPV1 lumbar versus sacral samples. A negative value for the FC indicates an upregulation in sacral DRG, whereas a positive FC indicates upregulation in lumbar DRG. Download Figure 4-1, XLSX file.

10.1523/ENEURO.0397-19.2019.f4-2 Extended Data Figure 4-2 Comparison of differential expression outcomes (lumbar vs sacral samples) between adult (control, i.e., total neuron population) and adult Trpv1 neurons. These lists show the genes that are differentially expressed between lumbar and sacral DRG in both groups (intersect), only differently expressed between lumbar and sacral DRG in the adult control group or only differently expressed between lumbar and sacral DRG in the adult Trpv1 group. These data are summarized in Venn diagram in Figure 4*C*. Download Figure 4-2, XLSX file.

This approach increased the sensitivity of the differential expression analysis, through the reduction of heterogeneity in the DRG starting material. Indeed, 6838 genes were differentially expressed between the Trpv1 sorted lumbar-sacral DEGs, almost 15 times more than we identified between the whole lumbar and sacral DRG tissue (*N* = 466; Fig. 4*B–D*; Extended Data Figs. 1-2, 4-1, 4-2). This allowed us to identify a very large number of novel genes upregulated in the sacral Trpv1 neurons, including a low-affinity nerve growth factor receptor Ngfr (alias p75), adenosine A1 receptor (Adora1), and Cacna2d2, an auxiliary subunit of a voltage-gated Ca channel targeted by the analgesics, pregabalin and gabapentin (Dolphin, 2016).

The majority of lumbar-sacral DEGs in the total DRG dataset were also differentially expressed between spinal levels in the Trpv1 population (Fig. 4*C*; Extended Data Fig. 4-2), with some exceptions including Htr1d, Kcng4, Ntrk3, Oprl1, Runx3, and Scrt1, which were all upregulated in lumbar DRG in the total adult sensory transcriptome but not differentially expressed across spinal levels of the Trpv1 dataset. This is consistent with Runx3 and Ntrk3 being primarily or solely expressed in large, myelinated, Trpv1-negative DRG neurons (Usoskin et al., 2015). The direction of the differential expression between lumbar and sacral DRG was the same in the total adult and Trpv1 neurons, with the exception of Ntrk2; this gene was upregulated in sacral DRG in the total DRG transcriptome but upregulated in lumbar DRG in the Trpv1 transcriptome. This could indicate that in sacral DRG Ntrk2 is more highly expressed in Trpv1-negative neurons.

**Figure 5.**
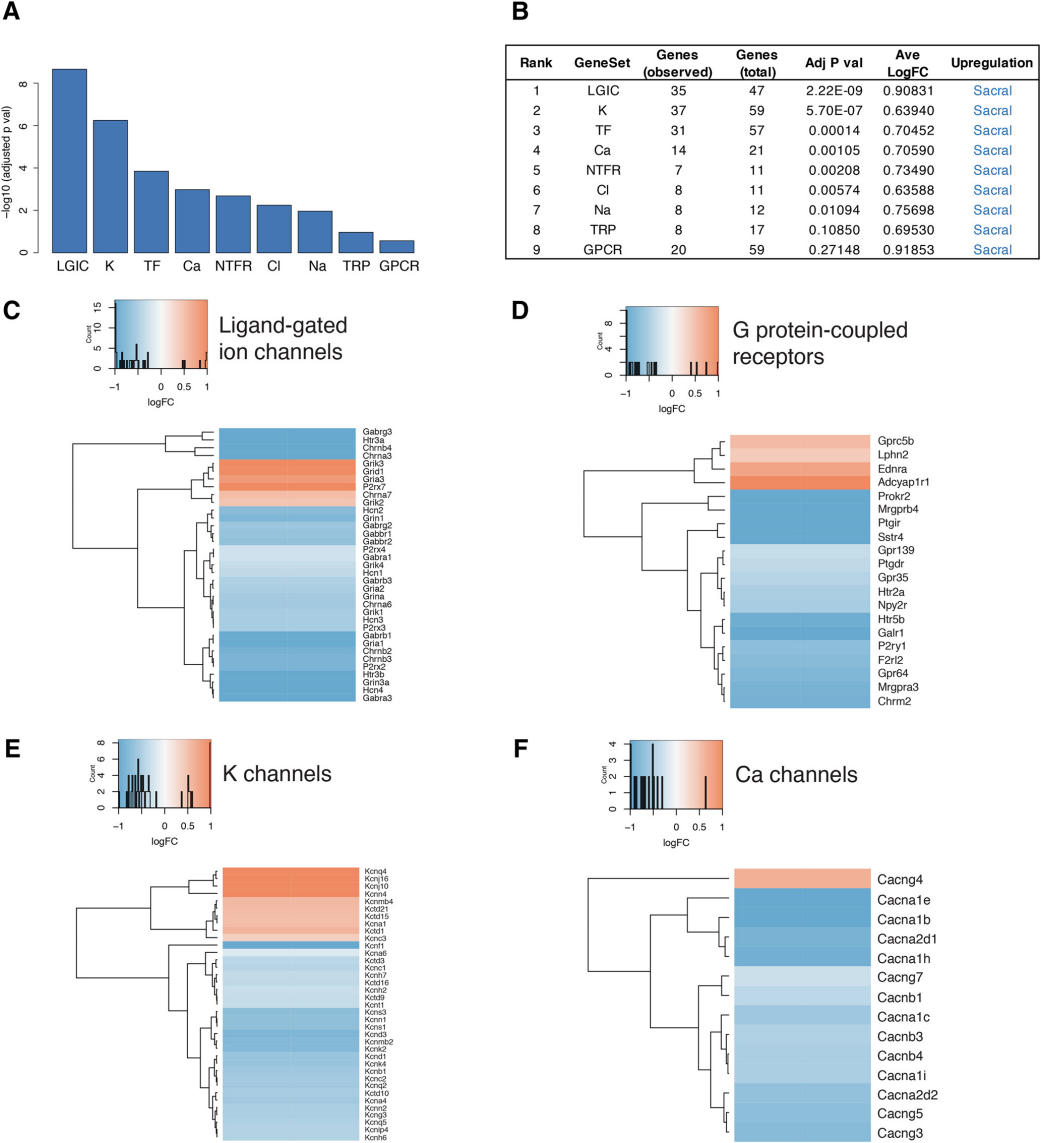
Gene set enrichment analysis of Trpv1-sorted DRG neurons taken from five male and five female adult mice, comparing expression at lumbar (L4-5) and sacral (L6-S1) levels. Nine of 10 gene sets (grouped according to shared function) were enriched (adjusted *p *<* *0.05): LGICs, TFs, GPCRs, NTFRs, K, Ca, and Cl channels, and TRP channels. ***A***, ***B***, All nine gene classes showed upregulation in sacral DRG (blue). ***C–F***, Genes differentially expressed between lumbar and sacral spinal levels from the LGIC, GPCR, K, and Ca channel classes. Blue indicates upregulation in sacral DRG and red upregulation in lumbar DRG. Full list of genes identified by this analysis is provided in Extended Data Figure 2-2, and a complete set of heatmaps is provided in Extended Data Figure 5-1.

More than 50% of the genes in our curated set (*N* = 298) were differentially expressed between Trpv1 lumbar and sacral DRG, most of which were upregulated in the sacral region (Fig. 5; Extended Data Fig. 4-1). This observation is consistent with a high proportion of sacral-specific genes being expressed in Trpv1 neurons. Gene set enrichment analysis of our curated sets indicated nine out of 10 gene sets were enriched, all of which showed upregulation in the sacral region (Fig. 5; Extended Data Figs. 2-2, 5-1). LGICs, K channels, GPCRs and TFs were among the largest groups differentially expressed. For example, genes associated with the LGIC set included many genes relating to glutamate and GABA signaling, the ionotropic serotonin receptors Htr3a and Htr3b, nicotinic cholinoceptor subunits (Chrna3, Chrna6, Chrnb2, Chrnb3, Chrnb4), hyperpolarization-activated cyclic nucleotide-gated channels (Hcn1, Hcn2, Hcn3, Hcn4) and purinoceptors (P2rx2, P2rx3, P2rx4). Genes associated with the GPCRs included serotonin receptors (Htr2a and Hrt5b), neuropeptide receptors (Galr1, Npy2r, Sstr4), the purinoceptor P2ry1 and muscarinic cholinoceptor, Chrm2, were also upregulated in sacral DRG. Taken together, the molecular profile indicates our method for isolating Trpv1 positive neurons provides an unprecedented opportunity to identify novel sacral-specific targets for nociceptive modulation.

10.1523/ENEURO.0397-19.2019.f5-1 Extended Data Figure 5-1 Gene set enrichment analysis of Trpv1-sorted DRG neurons taken from five male and five female adult mice, comparing expression at lumbar (L4-5) and sacral (L6-S1) levels. Nine of 10 gene sets (grouped according to shared function; see Extended Data Fig. 2-1) were enriched (adjusted *p *<* *0.05): LGICs, TFs, GPCRs, NTFRs, K, Ca and Cl channels, and TRP channels. Heatmaps show genes differentially expressed between lumbar and sacral spinal levels from each class. Blue indicates upregulation in sacral DRG and red upregulation in lumbar DRG. A list of genes identified by this is analysis provided in Extended Data Figure 2-2. Download Figure 5-1, PDF file.

### Many but not all aspects of differential gene expression between lumbar and sacral DRG are established by E18.5

**Figure 6.**
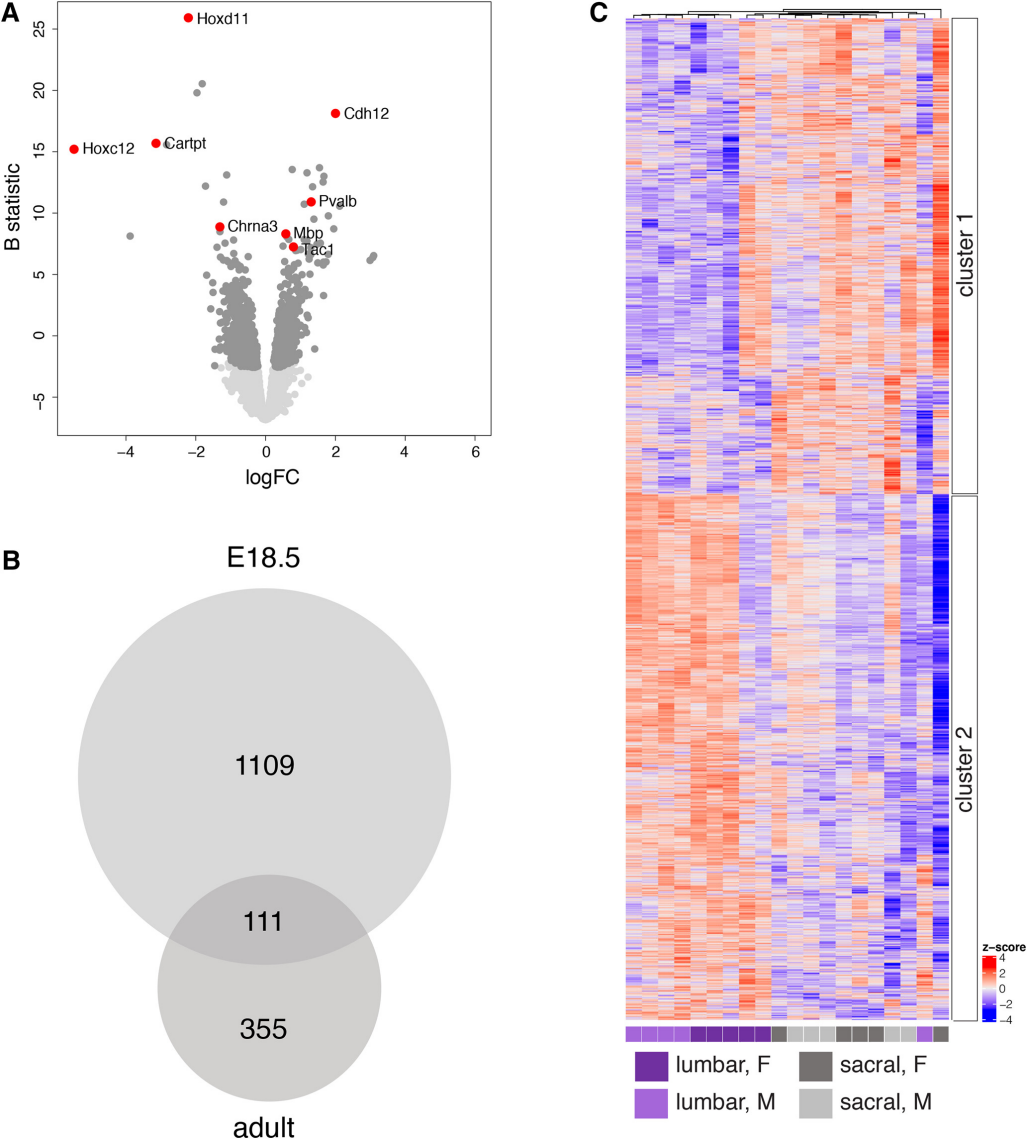
Analysis of DRG from two different spinal levels (lumbar: L4-5; sacral: L6-S1) taken from five male and five female E18.5 mouse embryos. ***A***, Volcano plot illustrating genes differentially expressed between lumbar and sacral DRG neurons. Light gray points are genes not differentially expressed, dark gray points are differentially expressed at adjusted *p *<* *0.05 (*N* = 466); examples of specific differentially expressed genes are highlighted in red. A negative value for the FC indicates an upregulation in sacral DRG, whereas a positive FC indicates upregulation in lumbar DRG. Full dataset provided in Extended Data Figure 6-1. ***B***, Proportional Venn diagram showing the number of genes differentially expressed between spinal levels (pooled male and female data), in the total E18.5 and total adult DRG population. A total of 466 genes were differentially expressed between lumbar and sacral levels in the total population of adult DRG neurons (adjusted *p *<* *0.05), 111 of which were also detected as differentially expressed between lumbar and sacral levels in E18.5 DRG neurons. An additional 1109 genes were detected as differentially expressed between lumbar and sacral levels only in the E18.5 DRG population. Gene lists summarized in Venn diagram are provided in Extended Data Figure 6-2. ***C***, Heatmap with hierarchical cluster for all differentially expressed genes (*N* = 1220; adjusted *p *<* *0.05) for E18.5 lumbar and sacral samples (*N* = 10). Both samples and rows are clustered using Pearson correlation. Heat color reflects row-wise *z* score, and samples are colored according to spinal level and sex. Ranked gene lists for heat map (clusters 1 and 2) are provided in Extended Data Figure 1-2.

A comparison of transcriptomes and lumbar-sacral differential gene expression between maturational states indicated that while some features of nociceptor and proprioceptor transcriptomes were already present at E18.5, DRG continue to develop the more complex expression profiles established in the adult (Fig. 6; Extended Data Fig. 6-1). For example, primary molecular classifiers of eleven distinct classes of sensory neurons recently proposed in a single cell sequencing study (L4-6 spinal levels, adult mouse; Usoskin et al., 2015) were present in our adult DRG datasets and most were also present at E18.5. We further noted that at E18.5 there was no detectable expression of Sst, Mrgpra3, or Trpa1 (the latter previously reported to be expressed only from ∼P14; Hjerling-Leffler et al., 2007). Each of these have previously been linked to acute or chronic itch (Han et al., 2013; Wilson et al., 2013; Stantcheva et al., 2016; Huang et al., 2018), potentially reflecting a coordinated maturation of this behavior.

10.1523/ENEURO.0397-19.2019.f6-1 Extended Data Figure 6-1 Differential expression analysis for embryo (EXVIII.5) lumbar versus sacral samples. A negative value for the FC indicates an upregulation in sacral DRG, whereas a positive FC indicates upregulation in lumbar DRG. Download Figure 6-1, XLSX file.

10.1523/ENEURO.0397-19.2019.f6-2 Extended Data Figure 6-2 Comparison of differential expression outcomes (lumbar vs sacral samples) between embryonic (EXVIII.5) and adult neurons. These lists show the genes that are differentially expressed between lumbar and sacral DRG in both groups (intersect), differentially expressed between lumbar and sacral DRG only in the embryo group or differentially expressed between lumbar and sacral only in the adult group. These data are summarized in Venn diagram in Fig. 6*B*. Download Figure 6-2, XLSX file.

Maturational changes in the DRG sensory transcriptome were consistent with a higher proportional representation of myelinated neurons being established in lumbar ganglia by E18.5. Pooling data from male and female embryos, we identified 1220 genes differentially expressed between lumbar and sacral DRG (Fig. 6; Extended Data Fig. 6-1). Of these DEGs, 111 were also differentially expressed between spinal levels in the adult (Fig. 6*B*; Extended Data Fig. 6-2). These include: Cartpt, Chrna3, Chrnb4, Esr1, Gabrg3, Gabra3, Htr3a, and Trpv1 that were upregulated in sacral DRG; Nefh and Pvalb were upregulated in lumbar DRG. However, many of the lumbar-sacral DEGs identified in the adult were not identified as differentially expressed between spinal levels in the embryo, indicating further maturation of some cell types after birth. These include: Calca, Chrnb3, Gap43, Galr1, Gfra3, Ntrk2, and Th upregulated in adult sacral DRG and Chrna7, Mpz and Runx3 upregulated in adult lumbar DRG. Tac1, identified in our adult DRG study as upregulated at the sacral level in adult mice, showed a small upregulation at the lumbar level in E18.5 DRG, further demonstrating that some aspects of nociceptor phenotype are yet to emerge in sacral nociceptors at this time. We also identified numerous lumbar-sacral DEGs (1109 genes) at E18.5 that were not detected as lumbar-sacral DEGs in adult DRG. These included: Calb1, Ntn1, Snai2, and Sstr4 upregulated in sacral DRG; Htr2c, Trpm8, Bdnf, Nos1, and Chrm2 upregulated in lumbar DRG. Together, this maturational analysis further demonstrates the dynamic nature of sensory neuron phenotypes in the perinatal and postnatal periods (Rifkin et al., 2000; Hjerling-Leffler et al., 2007; Isensee et al., 2017).

### Sex differences in the DRG transcriptome are more prevalent in sacral Trpv1 neurons

**Figure 7.**
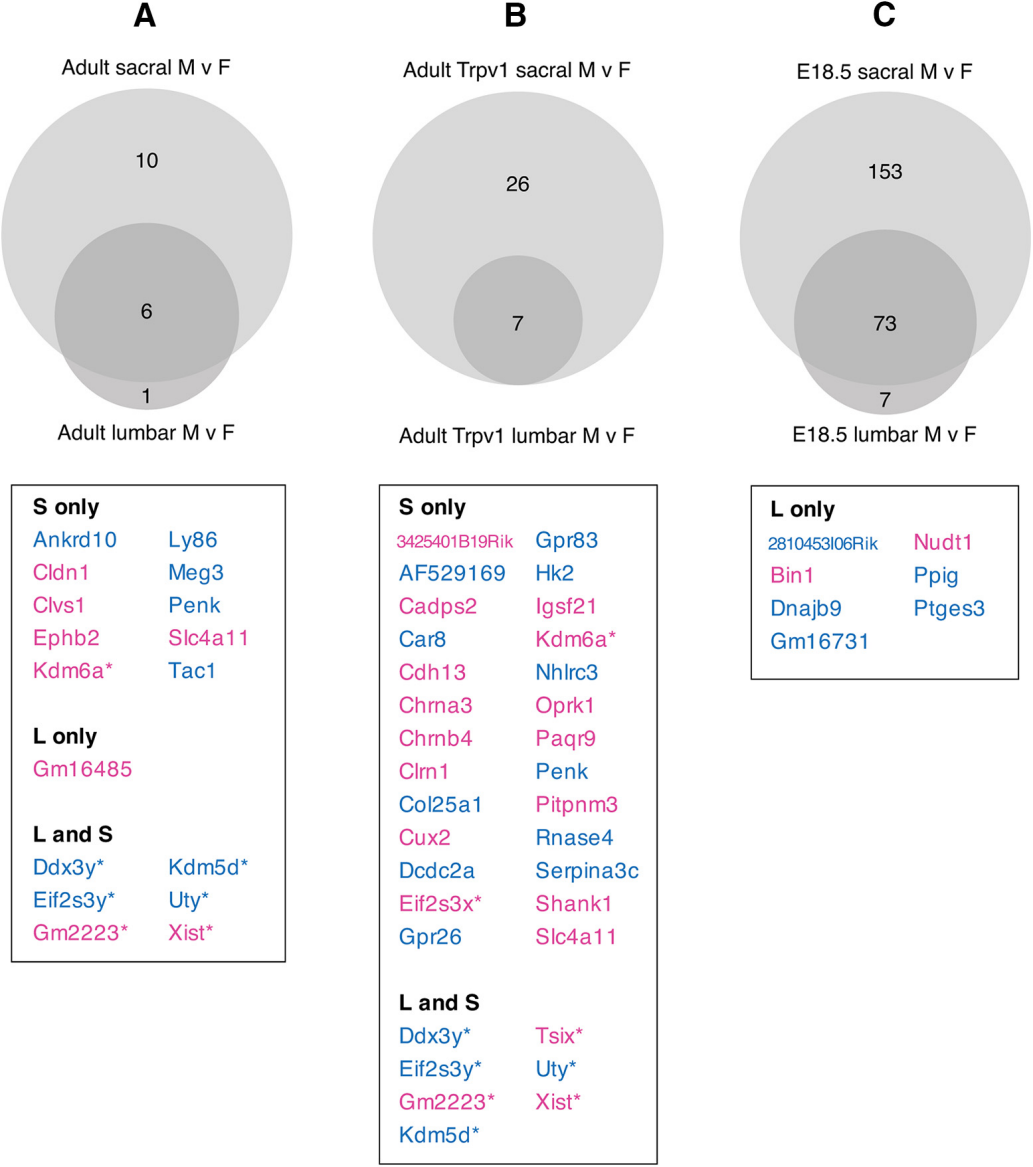
Sex differences in sensory neurons. Proportional Venn diagrams indicate the number of genes differentially expressed (adjusted *p *<* *0.05) between male and female groups in each spinal region of adult (***A***), Trpv1 (***B***), and E18.5 (***C***) populations. ***A***, In adult DRG, 17 genes were detected as differentially expressed between males and females; of these, 10 were differentially expressed only in sacral DRG, one only in lumbar DRG and six were differentially expressed between males and female DRG of both lumbar and sacral spinal levels. ***B***, In the Trpv1 adult DRG neurons, 33 genes were detected as differentially expressed between males and females; of these, 26 were differentially expressed between sex only in sacral DRG, seven were differentially expressed between sex in DRG of both spinal levels and no genes were differentially expressed between sexes only at the lumbar level. ***C***, In E18.5 DRG neurons, 233 genes were detected as differentially expressed between males and females; of these, 153 were differentially expressed between sex only in sacral DRG, seven only in lumbar DRG and 73 were differentially expressed between sex at both spinal levels. Where <30 genes were identified as differentially expressed between males and females, the specific genes are listed in the table below the relevant Venn diagram. These lists distinguish genes differentially expressed only in the lumbar or sacral levels, or differentially expressed in both levels. Genes listed in blue were upregulated in males, whereas genes listed in pink were upregulated in females. Asterisks indicate genes located on sex chromosomes. Full lists of differentially expressed genes for ***A–C*** are found in ***E***. Full gene lists for ***A–C*** are provided in Extended Data Figures 7-1, 7-2, 7-3.

To evaluate sex differences in the molecular profile of visceral sensory neurons we compared male and female samples from each DRG region in the adult population, Trpv1 population, and E18.5 population (Fig. 7). A recent study of lumbar (L1-5) DRG in adult mice identified a small number of genes differentially expressed between males and females, mainly genes located on sex chromosomes (Lopes et al., 2017b). Our analyses of adult DRG identified a similar group of genes that were differentially expressed between male and female DRG at both spinal levels (Xist, Ddx3y, Uty, Eif2s3y, Kdm5d, Gm2223; Fig. 7*A*; Extended Data Figs. 7-1, 7-2, 7-3). However, in adult sacral DRG, we identified an additional 10 genes differentially expressed between males and females (Fig. 7*A*). One of these genes (Kdm6a) is commonly identified in studies of sex differences (Arnold et al., 2016; Balaton and Brown, 2016), but several others have not previously been revealed in studies of sexual dimorphism in nociception. Some of these genes, including Penk and Tac1, are clearly neuronal, but the cellular origin of others remains to be defined. When separately considering the adult Trpv1 dataset, we detected several additional genes that were differentially expressed between males and females, all except one of which were identified only in sacral DRG (Fig. 7*B*; Extended Data Fig. 7-3). These included the nicotinic cholinoceptor subunits Chrna3 and Chrnb4, a membrane progestin receptor Paqr9 and several GPCRs.

10.1523/ENEURO.0397-19.2019.f7-1 Extended Data Figure 7-1 Differential expression analysis for male versus female adult lumbar samples. A negative value for the FC indicates an upregulation in male DRG, whereas a positive FC indicates upregulation in female DRG. Download Figure 7-1, XLSX file.

10.1523/ENEURO.0397-19.2019.f7-2 Extended Data Figure 7-2 Differential expression analysis for male versus female adult sacral samples. A negative value for the FC indicates an upregulation in male DRG, whereas a positive FC indicates upregulation in female DRG. Download Figure 7-2, XLSX file.

10.1523/ENEURO.0397-19.2019.f7-3 Extended Data Figure 7-3 Sex differences in adult, Trpv1 and embryo datasets. For each set of data (adult, Trpv1, embryo), these lists show the genes that are differentially expressed between male and female DRG only in the lumbar DRG (lumbar), only in the sacral DRG (sacral), or in both lumbar and sacral DRG (intersect). These data are summarized in Venn diagrams in Figure 7. Download Figure 7-3, XLSX file.

We then asked whether these sex differences were evident in embryonic DRG, given that sex organ development is not complete and exposure to sex steroids is more limited. The overall differences between genes upregulated in lumbar (E18.5 *N* = 80 adjusted *p *<* *0.05) and sacral (E18.5 *N* = 226 adjusted *p *<* *0.05) populations persisted (Fig. 7*C*; Extended Data Fig. 7-3). Of these genes differentially expressed between sexes at E18.5, 73 genes were in common to both lumbar and sacral DRG. These included similar groups of X-linked or Y-linked genes identified in adults.

## Discussion

**Figure 8.**
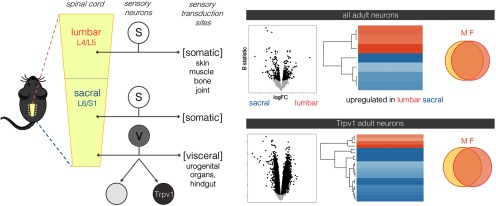
Summary of experimental outcomes in adult mice. Sensory neurons from DRG at different spinal levels contain either entirely somatic sensory neurons (S) or a mixture of somatic and visceral (V) neurons. Genes differentially expressed between DRG at these spinal levels indicate features of pelvic visceral sensory neurons and are represented here by volcano plots (all genes) and heat maps (GPCRs). Very few genes are differentially expressed between males and females. Isolation of Trpv1-expressing nociceptors provides a more sensitive assay for revealing genes differentially expressed between spinal levels (all genes and GPCRs shown as per upper panel) and sex differences.

We have identified numerous genes that are differentially expressed between lumbar and spinal DRG and point to a unique phenotype of the sensory neuron population that innervates pelvic viscera. Many but not all of these genes are already differentially expressed at embryonic day 18. By applying a novel approach for isolating Trpv1 neurons, we identified patterns of differential expression with a higher level of resolution and revealed sex differences in gene expression that were not detected across the total population of sensory neurons (Fig. 8). Together, our datasets have the potential to provide new insights into the specific features of pelvic visceral sensory neurons that underlie their unique behaviors.

Several publications have provided deep insights into the complexity of the sensory neuron transcriptome (Lerch et al., 2012; Manteniotis et al., 2013; Chiu et al., 2014; Goswami et al., 2014; Thakur et al., 2014; Reynders et al., 2015; Usoskin et al., 2015; Lopes et al., 2017a; Ray et al., 2018), however to our knowledge none have yet focused on the total population of neurons innervating pelvic viscera. Our goal was not to identify specific cell classes or quantify expression in individual neurons but to define gene expression patterns distinctive for spinal level, sex and maturational state. Our data point to specific novel gene sets to guide focused quantitative studies on expression in individual cell types. By selecting DRG from the L6-S1 spinal levels, we deliberately biased our detection of differentially expressed genes to neurons that are directly involved with the sensation in pelvic viscera; however, we also recognize that somatic afferents present in these sacral DRG may exhibit differences from lumbar DRG and contribute to differentially gene expression across spinal levels.

Visceral sensory neurons have many properties that differ from somatic sensory neurons and that are relevant to their distinct responses to analgesics, responses to injury and other pathophysiological perturbations (Robinson and Gebhart, 2008; Schwartz and Gebhart, 2014; Gebhart and Bielefeldt, 2016; Grundy et al., 2019). Our analyses of aggregated pelvic visceral sensory neurons provide the opportunity to understand the molecular contributors to these distinctive features that are shared by this population. Our approach aimed to identify the transcriptome underlying unifying features of pelvic visceral sensory neurons, aligned with their common physiological and pathophysiological features. A logical extension of this would be to characterize the transcriptome of sensory neurons innervating particular a specific organ or perturbation of interest, by isolating neurons that have been labeled with conventional retrograde tracer applied to that site (Hockley et al., 2019). This has not yet been performed across the many types of pelvic organs.

A previous study to characterize the transcriptome of DRG neurons projecting to the distal colon in adult mice (Hockley et al., 2019) provides an important dataset for the field of visceral sensory neurobiology and pathophysiology. As expected, there is a strong congruence between the gene sets identified in colonic afferents and our data that we considered to represent the total sacral visceral population. For example, each of the genes considered as primary classifiers for the seven clusters of colonic afferents is also expressed in our male and female sacral datasets. However, from these data, we are not yet able to determine if afferents innervating the reproductive or lower urinary tracts show different expression profiles to isolated colonic afferents. This is largely because levels of gene expression cannot be directly compared across single cell and bulk sequencing studies. For example, from our current data, we cannot validly deduce clusters of genes that represent cell types. We also cannot estimate the proportion of neurons that coexpress particular gene combinations. Many neurons show functional phenotypic differences due to gene combinations or levels of protein expression rather than absolute expression levels of a single gene. We further note that the two studies were designed to compare gene expression across spinal levels for different purposes – our study compared L6-S1 (mixed visceral-somatic) with L4-5 (purely somatic) ganglia, whereas Hockley et al. (2019) characterized visceral neurons that innervate the distal colon, comparing neurons from T10-L1 and L5-S2. Finally, their study focused only on males, whereas our study separately collected data from male and female mice.

We have not attempted to provide a complete investigation of each gene differentially expressed across lumbar and sacral spinal levels, but instead focused on several genes relevant to neural signaling. We found a particularly interesting aspect of expression of the nicotinic cholinoceptor subunits, Chrna3 and Chrnb4, that showed a profile distinct from other nicotinic receptor genes. Both were upregulated in E18.5 and adult sacral DRG where no differential expression between sexes was identified. However, in the adult Trpv1 subpopulation we identified higher expression of both genes in the sacral DRG and a sex difference (upregulation in female). Three other nicotinic subunits (Chrna6, Chrnb2, and Chrnb3) were also upregulated in sacral Trpv1 neurons, but no sex differences were observed. Historically, the primary focus of nicotinic receptor characterization in the peripheral nervous system has been ganglionic transmission in autonomic circuits, where the predominant subunit combinations of nicotinic receptors are α3β4 and α3β5β4 (McGehee and Role, 1995). However, nicotinic receptors have also been reported in sensory neurons (Genzen et al., 2001; Rau et al., 2005), including bladder-projecting sacral DRG (Nandigama et al., 2013). A recent study has shown that Chrna3 is more highly expressed in visceral sensory neurons, including neurons that innervate the bladder and colon, and that it distinguishes “silent” peptidergic nociceptors that become sensitized to mechanical stimuli during inflammation (Prato et al., 2017). Beyond being a marker for a functionally distinct class of sensory neurons, there are several possible sources of endogenous acetylcholine that may modulate sensory activity by activating these nicotinic receptors. These include macrophages (Fujii et al., 2017) and nearby cholinergic autonomic axons. Our data revealing sex differences in the expression of Chrna3 and Chrnb4 by sacral Trpv1 neurons raises the possibility of targeting these channels to modulate sacral visceral pain in females. This sensory context of nicotinic function also provides a new perspective to previous studies where several types of nicotinic receptor gene deletion (including Chrna3 or Chrnb4) impaired urinary voiding (Xu et al., 1999a,b); these mice exhibiting enlarged bladders, “dribbling urination,” and urothelial hyperplasia. Although these animals clearly had parasympathetic deficits, a component of their overall phenotype may have been due to sensory dysfunction.

We have not attempted to distinguish neural and non-neural contributors to the differential gene expression patterns identified in our study. Many genes can be clearly attributed to neurons, glia or both, however a much greater proportion remain to be characterized in the context of the DRG. An intriguing example is the potent vasoconstrictor Uts2d that was expressed in adult DRG, and of all genes identified, had one of the highest FCs in expression across spinal levels (upregulated in sacral DRG). This gene was absent from our adult Trpv1 data. Uts2d was expressed in E18.5 DRG but did not show this differential expression across spinal levels. One interpretation is that Uts2d is expressed by Trpv1-negative neurons (or their associated glia) from E18.5 but that postnatally expression increases selectively in sacral Trpv1-negative neurons. The function of Uts2d is yet to be determined in sensory systems.

The TrpV1^PLAP-nLacZ^ mice used in our study on adult DRG have been extensively characterized and validated (Cavanaugh et al., 2011a,b) in anatomic and functional studies that show reporter expression to be a reliable indicator of adult Trpv1 expression (e.g., expression in around one-third of adult DRG neurons). In contrast, transcriptome profiles from adult Trpv1 DRG neurons have previously utilized Trpv1-Cre mice (Goswami et al., 2014) that overestimate the adult Trpv1 population because many immature DRG neurons express Trpv1 that is then downregulated postnatally (Cavanaugh et al., 2011a), i.e., the Cre positive neurons in the Trpv1-Cre mouse model will continue to identify the full Trpv1-Cre lineage rather than the exclusive adult Trpv1 expression. The approach we used to isolate Trpv1 neurons from adult mouse DRG was successful in providing a tool to analyze a Trpv1-enriched population, as determined by our assessment of expression patterns for genes known to be associated with major functional classes of DRG neurons. We were conservative in our cell sorting criteria (retaining only the brightest 15% of neurons) to reduce the prevalence of large myelinated neurons; the success of this is demonstrated by the expression profile of the Trpv1 flow cytometry output that shows low but not absent expression of key markers of these large myelinated neurons. Because of our conservative sorting criteria, we also predict that some of the Trpv1 neurons are missing from our data. Moreover, the neuronal isolation and flow cytometry protocols may have acutely affected transcription. These procedures were not applied to the total adult or E18.5 samples, limiting the direct comparison of expression levels that can be made across the sample sets.

Very few studies of the DRG transcriptome have directly compared males and females. A recent study by Lopes et al. (2017b) in adult mice identified a similar small group of X-linked and Y-linked genes, strongly overlapping with genes that we identified as differentially expressed between sexes. We did, however, identify a slightly larger group of differentially expressed genes than this study, likely because we included L6-S1 ganglia that innervate sexually dimorphic tissue. We detected the largest number of genes differentially expressed between males and females by restricting our analyses to Trpv1 neurons, consistent with these neurons having a strong innervation of the pelvic viscera. Several are genes that escape X inactivation (escape genes) that have been reported to show variable expression between tissue types (Arnold et al., 2016; Balaton and Brown, 2016; Tukiainen et al., 2017). The functional interpretation of this variability and its role in neural function is unclear but warrants further investigation.

In conclusion, we have identified numerous features of sensory neurons that vary between lumbar and sacral DRG and that are potentially involved in unique physiology and pathophysiology of visceral sensation and pain. There are limited sex differences across the transcriptome of sensory ganglia, but these can be revealed in sacral levels and especially in Trpv1 nociceptive neurons. These datasets will encourage identification of new tools to modify mature or developing sensory neurons and adult nociceptive pathways.

## Synthesis

Reviewing Editor: Quentin Pittman, University of Calgary Decisions are customarily a result of the Reviewing Editor and the peer reviewers coming together and discussing their recommendations until a consensus is reached. When revisions are invited, a fact-based synthesis statement explaining their decision and outlining what is needed to prepare a revision will be listed below. The following reviewer(s) agreed to reveal their identity: Jennifer DeBerry, Theodore Price. Note: If this manuscript was transferred from JNeurosci and a decision was made to accept the manuscript without peer review, a brief statement to this effect will instead be what is listed below.

Both reviewers and I agree that your data are solid, are potentially very interesting to the field and provide new information. However, there are several major issues that must be addressed:

1. Poor quality of the figures

2. Less than optimal way of presenting the data (as outlined in the reviews)

3. A somewhat superficial analysis

Specifically you must also make some comparison to the Smith and Bulmer Gut paper single cell date in order to really nail down the differences as due to transcriptomic differences of visceral projection neurons. On other words, a deeper discussion of the single cell data is warranted

The figures should include some sort of label for directionality of expression change, whether or not the gene is sex-linked, and for lists that are less than 50 or so genes long should not be cherry picked. The sex difference figures is the most obvious one that can be more informative.

Please also insure that your data are available on public hosting sites such as gudmap.

Additional specific suggestions for revision can be found in the individual reviews appended below:

Reviewer 1:

This manuscript provides a host of mouse transcriptome data that is informative to a range of neuroscience disciplines, particularly to the field of visceral sensory neuroscience. Specifically, the experimental design allows for determination of differentially expressed genes (DEGs) between predominately somatic versus somatic plus visceral DRG neurons, providing a clever way to circumvent the inherent problem in gene/protein expression studies of a characteristically small proportion of visceral neurons contained within sacral DRG. Data were generated from both male and female adult wild type animals to examine sex differences, which is certainly a clinically relevant factor given predominant sex differences in various visceral pain and function syndromes. Data were further refined by the use of a strategy involving DDAO expression, produced by cleaving of DDAOG by b-galactosidase (product of the lacZ gene), in Trpv1-PLAP-nlacZ mice. This largely enhanced the number of DEGs, and provides a plethora of information specifically related to nociceptive signaling. Finally, DRG taken at a late embryonic time point were sequenced for evaluation of developmental changes in segmental gene expression, again extending the impact of the data. To this reviewer’s knowledge, this is only the second report of transcriptome data for DRG neurons with specificity for visceral structures (a publication first released online in late 2018 describes single-cell sequencing data for colonic sensory neurons from adult male mice; Hockley et al. Gut 2019).

This manuscript provides a host of mouse transcriptome data that is informative to a range of neuroscience disciplines, particularly to the field of visceral sensory neuroscience. Specifically, the experimental design allows for determination of differentially expressed genes (DEGs) between predominately somatic versus somatic plus visceral DRG neurons, providing a clever way to circumvent the inherent problem in gene/protein expression studies of a characteristically small proportion of visceral neurons contained within sacral DRG. Data were generated from both male and female adult wild type animals, and were further refined by the use of a strategy involving DDAO expression, produced by cleaving of DDAOG by b-galactosidase (product of the lacZ gene), in Trpv1-PLAP-nlacZ mice. This largely enhanced the number of DEGs. Finally, DRG taken at a late embryonic time point were sequenced for evaluation of developmental changes in segmental gene expression. To this reviewer’s knowledge, this is only the second report of transcriptome data for DRG neurons with specificity for visceral structures (a publication first released online in late 2018 describes single-cell sequencing data for colonic sensory neurons; Hockley et al. Gut 2019). Overall the presentation of methods and data are clear, and enough information is provided for a similar analysis using R to be performed elsewhere. There are a few things that perhaps might be addressed more thoroughly. The authors describe a technical batch effect between experimental batches in the adult dataset, for which a software correction function was used. What was the outcome of the batch effect? and how was it determined to be a technical effect? Although the text sufficiently describes the R-based analysis steps, as noted below it is difficult to determine the rigor of statistical values given there is no information provided with respect to controlling false discovery rate. Figures are generally very informative, although in some places text (particularly gene names) are extremely small and very difficult to read. If the R version used allows for it, it would be highly interesting to include an analysis of alternative isoforms (for example using the JunctionSeq method), even if only in one experimental data set; however, it is understood that this may be beyond the scope of the current work.

Reviewer 2:

This work uses RNA seq on whole DRG and TRPV1-enriched populations of neurons between DRG levels that mostly innervate somatic tissues and visceral tissues. The logic underlying the experimental approach is sound and the TRPV1 neuronal population isolation method is clever. The sequencing appears to have been done well and the statistical analysis is adequate. My major issue with the paper is the way the findings are presented and the consideration of previous work. This paper could be an excellent resource for the field, but now I think the data presentation will be impenetrable to most readers.

1) The authors seem to be completely unaware of the previous work of Ewan St John Smith’s lab (Hockley et al, Gut 2019) where single cell RNA seq was done on colonic-projecting DRG neurons. This work needs to be included in the introduction and incorporated into the analysis. The authors do cite this paper in their discussion but only in passing. I really think that the authors should be using this dataset to reach conclusions about genes they are finding in their analysis. This would dramatically enhance the value of the paper.

2) The authors do not clarify whether their TRPV1 marking approach is driven by transcriptional programs occurring during development that would mark a larger proportion of neurons since TRPV1 is expressed in most sensory neurons during early development. This is mentioned in the methods, but the results do not demonstrate that the population of marked neurons is closer to 40% which would represent adult expression. This should be clarified.

3) The text in most of the figures is really small and hard to read. In Figure 2 C and D, what purpose do the very large bars in blue and orange serve? These should be smaller and the clustering and text should be larger. Overall, the data presentation is relatively poor in the paper.

4) The presentation of the data in Fig 7 is not informative. There are a relatively small number of genes shown in these Venn Diagrams (which are not proportional). This could be shown with directionality of the sex difference and all genes could be shown within the figure. Another issue is that it is not clear which of these genes are sex-linked and which are on other chromosomes. This should be shown in the figure.

5) The authors state that their study provides “new insight into specific features of pelvic visceral sensory neurons that underlie their unique behaviors and response to tissue damage” but I see very little evidence of such a claim. The nicotinic receptor features described here were already known and much of the other differences are not discussed at all. The analysis of the sex differences, which would be very interesting, is presented in a disorganized fashion that does not provide information to the reader. It is quite unfortunate that this rich dataset is presented in way that is really just scratching the surface of its value.
