# Supplementary figures and images for: Identification of a Sacral, Visceral Sensory Transcriptome in Embryonic and Adult Mice
Source: eNeuro. 2020 Feb 19;7(1):ENEURO.0397-19.2019. doi: 10.1523/ENEURO.0397-19.2019 (PMC7036621; doi:10.1523/ENEURO.0397-19.2019)

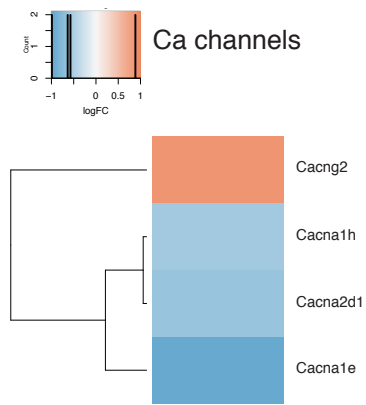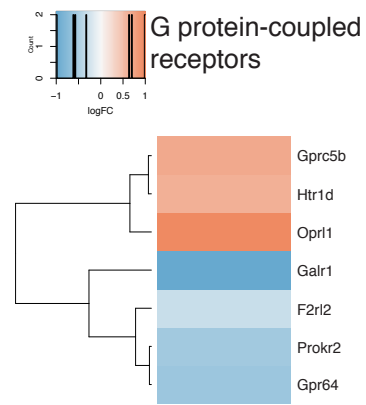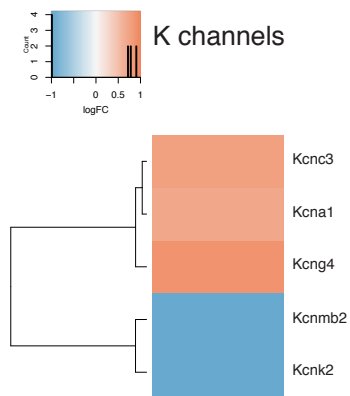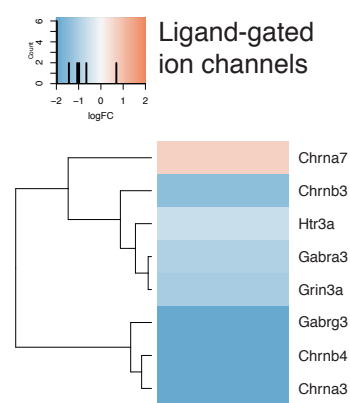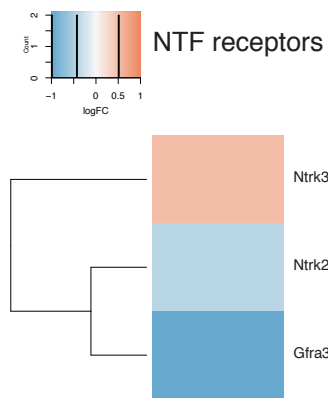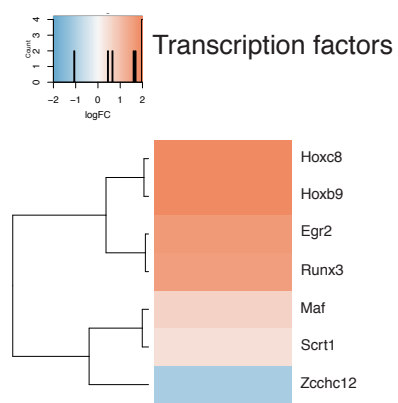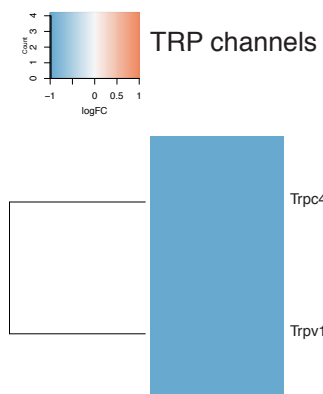

Supplement: Extended Data Figure 2-3 — Gene set enrichment analysis of DRG neurons taken from five male and seven female adult C57Bl/6 mice, comparing expression at lumbar (L4-5) and sacral (L6-S1) levels. Seven of 10 gene sets (grouped according to shared function; see Extended Data Fig. 2-1) were enriched (adjusted p < 0.05): LGICs, TFs, GPCRs, NTFRs, Ca and K channels, and TRP channels. Heatmaps show genes differentially expressed between lumbar and sacral spinal levels from each class. Blue indicates upregulation in sacral DRG and red upregulation in lumbar DRG. A list of genes identified by this analysis is provided in Extended Data Figure 2-2. Download Figure 2-3, PDF file. [file enu-eN-NWR-0397-19-s12.pdf]

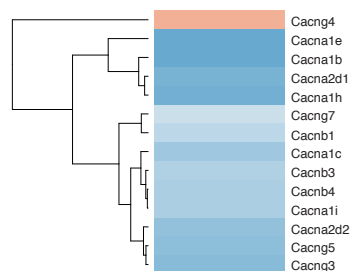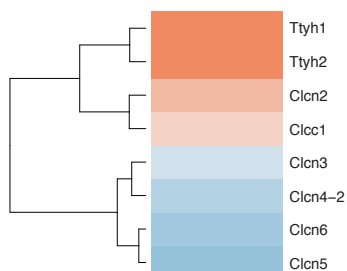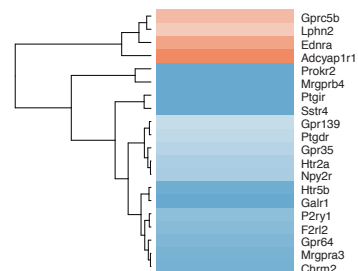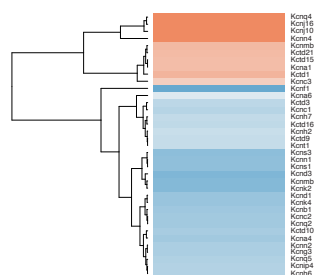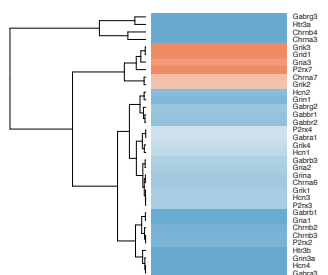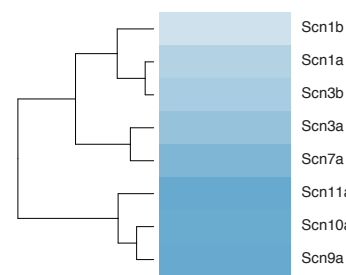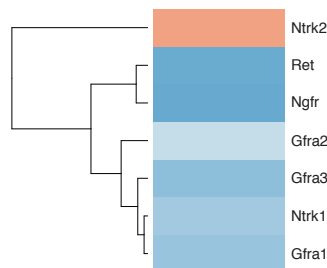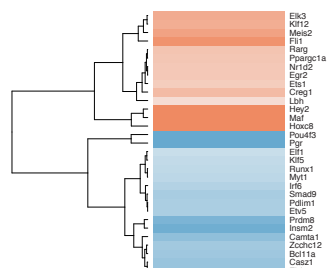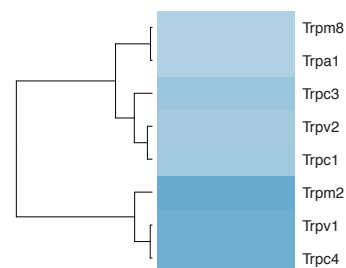

Supplement: Extended Data Figure 5-1 — Gene set enrichment analysis of Trpv1-sorted DRG neurons taken from five male and five female adult mice, comparing expression at lumbar (L4-5) and sacral (L6-S1) levels. Nine of 10 gene sets (grouped according to shared function; see Extended Data Fig. 2-1) were enriched (adjusted p < 0.05): LGICs, TFs, GPCRs, NTFRs, K, Ca and Cl channels, and TRP channels. Heatmaps show genes differentially expressed between lumbar and sacral spinal levels from each class. Blue indicates upregulation in sacral DRG and red upregulation in lumbar DRG. A list of genes identified by this is analysis provided in Extended Data Figure 2-2. Download Figure 5-1, PDF file. [file enu-eN-NWR-0397-19-s13.pdf]
